# Analysis of repeat elements in the *Pristionchus pacificus* genome reveals an ancient invasion by horizontally transferred transposons

**DOI:** 10.1186/s12864-022-08731-1

**Published:** 2022-07-19

**Authors:** Marina Athanasouli, Christian Rödelsperger

**Affiliations:** Max Planck Institute for Biology, Department for Integrative Evolutionary Biology, Max-Planck-Ring 9, 72076 Tübingen, Germany

**Keywords:** Evolution, Nematode, Comparative genomics, *Caenorhabditis elegans*, Repetitive, Zisupton

## Abstract

**Background:**

Repetitive sequences and mobile elements make up considerable fractions of individual genomes. While transposition events can be detrimental for organismal fitness, repetitive sequences form an enormous reservoir for molecular innovation. In this study, we aim to add repetitive elements to the annotation of the *Pristionchus pacificus* genome and assess their impact on novel gene formation.

**Results:**

Different computational approaches define up to 24% of the *P. pacificus* genome as repetitive sequences. While retroelements are more frequently found at the chromosome arms, DNA transposons are distributed more evenly. We found multiple DNA transposons, as well as LTR and LINE elements with abundant evidence of expression as single-exon transcripts. When testing whether transposons disproportionately contribute towards new gene formation, we found that roughly 10–20% of genes across all age classes overlap transposable elements with the strongest trend being an enrichment of low complexity regions among the oldest genes. Finally, we characterized a horizontal gene transfer of Zisupton elements into diplogastrid nematodes. These DNA transposons invaded nematodes from eukaryotic donor species and experienced a recent burst of activity in the *P. pacificus* lineage.

**Conclusions:**

The comprehensive annotation of repetitive elements in the *P. pacificus* genome builds a resource for future functional genomic analyses as well as for more detailed investigations of molecular innovations.

**Supplementary Information:**

The online version contains supplementary material available at 10.1186/s12864-022-08731-1.

## Background

Repetitive DNA describes sequence motifs repeated from hundreds to thousands of times within a genome. Repetitive DNA represents a large fraction of eukaryotic genomes, hampering genome assembly and annotation [1]. The fraction of repetitive sequences in a genome varies across species, from 12% in *Caenorhabditis elegans* to 80% in some plants [2, 3]. While their role and significance is not fully understood, the origin for the majority of these repeats has been traced to transposable elements (TEs) due to their mobility and their ability to increase their copy-number rapidly [2, 4]. The current hierarchical classification system for TEs was proposed by Wicker et al. in 2007 and takes into consideration the structural characteristics of TEs as well as their mode of replication [5]. Based on this system, TEs are classified into retrotransposons utilizing a RNA intermediate for mobilization (class I) and transposons with a DNA intermediate (class II). Class I TEs are further divided into Long Terminal Repeats (LTRs) and non-LTR sequences while class II includes DNA and rolling circle (RC) elements. Initially labelled as selfish elements, TEs have been linked to metazoan genome evolution and regulation of processes associated with development and diseases [5, 6].

Until now, little is known about the impact of TEs in nematode genome evolution. In plant-parasitic nematodes, the high frequency of TEs has been associated with polyploidy and is thought to affect genome adaptation [7–9]. In *C. elegans* where 12% of the genome is estimated to be covered by TEs, experimental evidence of transposon activity is sparse with the exception of the DNA transposon superfamily Tc1/Mariner [10]. The free living nematode *Pristionchus pacificus* was introduced as a satellite model organism to *Caenorhabditis elegans* but has since been established as an independent model organism for studying phenotypic plasticity and genome evolution due to novel traits not observed in *C. elegans* [11–13]. *P. pacificus* has an established genetic toolkit and a chromosome-scale genome assembly [14]. The combination of comparative genomics and subsequent manual curation of the gene predictions produced by automated pipelines has generated a high quality gene annotation for *P. pacificus* [15, 16]. However, the current annotation does not include a dataset for repetitive sequences and specifically TEs. A comprehensive characterization of repetitive sequences in *P. pacificus* is of particular importance for us as this may complement current studies to understand the origin and evolution of new genes [17]. Previous studies have shown that new genes show substantial contributions by transposons [18]. In addition, a recent study in *P. pacificus* demonstrated that repetitive sequences can cause homology detection failures leading to misclassifications as species-specific genes [19]. Therefore, we want to test whether new genes preferentially show overlaps with such repetitive sequences.

In this study, we provide the first complete repeat dataset for *P. pacificus*. To that end, we applied different approaches to identify and annotate repetitive sequences in this nematode. The resulting datasets were evaluated and RepeatModeler2 was chosen for further analysis due to the high coverage, agreement with the other methods, as well as the TE classification it provides. Subsequently we utilized the available transcriptomic, phylostratigraphic and gene annotation data to screen for evidence of active transcription of TEs. We found multiple candidates for active transposons while simple repeats were overrepresented in protein-coding genes. Contrary to our expectation, we do not see a strong trend towards an enrichment of repetitive elements among young genes. We actually found an opposing trend with the strongest signal being an overrepresentation of simple repeats among old gene classes. Finally, we identified distant homologs of the Zisupton DNA transposon superfamily which is absent in *C.elegans* but present in fishes, fungi and other metazoans and attributed their presence in *P. pacificus* to horizontal gene transfer.

## Results

### There is little agreement between different repeat finders

**Fig. 1:**
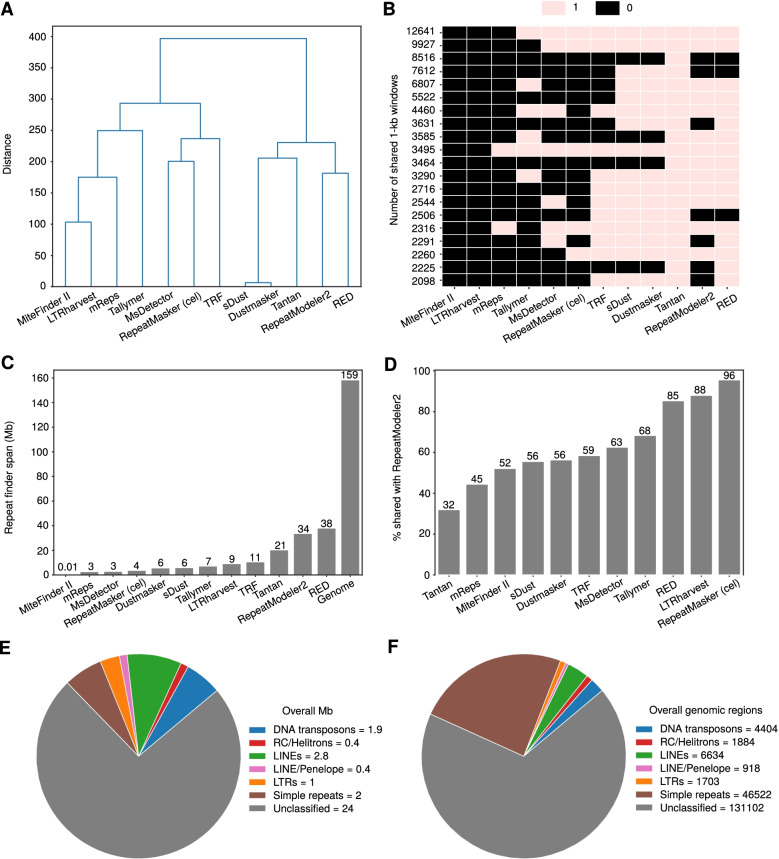
**A** Hierarchical clustering of the tools used for de novo repeat detection in *P. pacificus* based on 1-kb non-overlapping windows identified repeat finders with similar performance. The y-axis reflects the Euclidean distance between the binary vectors of the 1-kb windows. **B** The 20 most abundant patterns of common 1-kb windows reflected the clustering results. **C** The sum of nucleotides each repeat finder spanned in Mb was calculated and compared to the entire genome of *P. pacificus*. RED masked the most genomic regions with RepeatModeler2 following. **D** The comparison between each of the repeat finders and RepeatModeler2 showed that the level of agreement varies, ranging from 95.6% with RepeatMasker (*C. elegans* as a template) to 32% with Tantan. **E** The pie chart shows the amount of repetitive regions identified by RepeatModeler2. Class I TEs and specifically LINEs, LTRs and Penelope elements consist the majority of annotated TEs in the *P. pacificus* genome. **F** The pie chart shows the number of identified RepeatModeler2 regions for each type. The majority of annotated masked genomic regions is assigned to simple repeats

We initially used RepeatMasker (A.F.A. Smit, R. Hubley & P. Green RepeatMasker, http://repeatmasker.org) with *C. elegans* as a reference to identify repeats in *P. pacificus*. This approach masked 3.8 Mb (2.4%) of the *P. pacificus* genome, a small portion considering the fact that *P. pacificus*’ genome is larger than *C. elegans*’ combined with previous knowledge regarding the amount of repetitive elements in *C. elegans* [3, 10]. The failure of RepeatMasker could be attributed to divergence between the two genomes and horizontal gene transfer which deemed *C. elegans* an insufficient reference and de novo repeat detection necessary. For this purpose, we chose 11 additional tools representing a variety of approaches, ranging from de novo identification based on machine-learning to library-based detection [20–30]. The repeat finders applied to this study are listed in Table 1. To compare methods, we split the *P. pacificus* genome in consecutive 1-kb windows. We then encoded repeat information as 1 if a 1-kb window contained repeats by a given method and 0 otherwise. Subsequently we used hierarchical clustering and analysis of most abundant patterns to compare different methods. Similar approaches clustered together as is the case for the RED/RepeatModeler2 and Dustmasker/sDust pairs (Fig. 1A). Furthermore, software tools like MiteFinderII and mReps which masked a small percentage of the genome were separated from the other tools, an indication of lower effectiveness in identifying TE in the nematode’s genome. This is mirrored in the most abundant patterns of 1-kb windows (Fig. 1B). Out of the 117,134 most common 1-kb windows between the different tools, RepeatModeler2 and RED shared the majority (*N* = 77,679 1-kb windows) while all of the approaches except LTRharvest, MiteFinderII and mReps shared the top 12,641 1-kb windows (Fig. 1B). LTRharvest and MiteFinderII regions were completely absent throughout the most abundant patterns (Fig. 1B). On the contrary, coverage by Tantan was present in all windows of the presence/absence heatmap which spans approximately 123 Mb. These comparisons revealed little congruence between the different datasets and we thus wanted to decide which dataset to use for further analysis.

**Table 1:** The repeat finders used to detect repeat sequences incorporate a variety of approaches, ranging from library-based masking (e.g. RepeatMasker) to machine-learning using the reference genome (e.g. RED)

Software	Type of repeat	Method	References
RED	Tandem repeats, TEs	Machine-learning	[20]
LTRharvest	Long terminal repeat retrotransposons	Signature-based	[21]
Tallymer	Tandem repeats, TEs (plants)	De novo based on k-mers	[22]
MiteFinderII	Miniature inverted repeat TEs	De novo based on k-mers	[23]
TRF	Low-complexity regions, tandem repeats	De novo	[24]
Tantan	Low-complexity regions, short tandem repeats	De novo	[25]
MsDetector	Microsatellites	Learning-based	[26]
RepeatModeler2	Tandem repeats, TEs	Consensus	[27]
sDust	Low-complexity regions, tandem repeats	De novo	[28]
Dustmasker	Low-complexity regions, tandem repeats	De novo	[29]
mReps	Low-complexity regions, tandem repeats	De novo	[30]
RepeatMasker	Tandem repeats, TEs	Library-based	A.F.A. Smit, R. Hubley & P. Green RepeatMasker, http://repeatmasker.org

### The RepeatModeler2 dataset provides high coverage and TE annotation

To decide which repeat annotation to accept as the most representative, we considered the total genomic coverage for each method. We elected a baseline equal to 12% of the genome due to the repetitive content of *C. elegans.* This threshold is the lower estimate regarding the span of repeats in nematodes as shown in relevant work [4, 9, 31, 32]. For a comprehensive annotation we arbitrarily decided to accept possible false positives and therefore focus on approaches which identified around 12% or even a larger fraction of the *P. pacificus* genome as repetitive. Compared to the other methods, RED masked the highest portion of the genome with 38.2 Mb of repeats identified, closely followed by RepeatModeler2 (Fig. 1C). On the contrary, MiteFinderII and mReps produced low coverage masking data. We performed pairwise comparisons for the masked regions between each of the other software tools and RepeatModeler2 to determine the level of agreement between RepeatModeler2 and the remaining methods. As expected, the RepeatModeler2 dataset incorporated almost all the genomic loci identified by RepeatMasker (using the *C. elegans* repeat library available) and the majority of the repeats identified by RED and LTRharvest (Fig. 1D). RepeatModeler2’s dataset was chosen for further investigation due to the high agreement with the majority of the approaches as well as the annotation it provides, with 9.8 Mb out of the total 33.8 classified (Fig. 1E).

### Retrotransposons are the most abundant TE class, accounting for 50% of annotated repeats

According to the RepeatModeler2 classification, the most abundant repetitive sequences in the *P. pacificus* genome are Long Interspersed Nuclear Elements (LINEs), with simple repeats and DNA transposons following closely (Fig. 1E, F). Penelope elements, LTRs and RC/Helitrons make up a small portion of the RepeatModeler2 dataset. It is worth noting that RepeatModeler2 could not distinguish Penelope elements from LINEs, offering instead a unified classification as Penelope/LINE. RepeatModeler2 did not annotate any of the identified repetitive sequences as a SINE, in contrast to RepeatMasker which yielded 30 SINEs with *C. elegans* as a reference. In order to improve classification, we tested DeepTE [33] and the RFSB classifier from transposonUltimate [34] against the annotated dataset of RepeatModeler2. Both classifiers differed from the RepeatModeler2 homology-based classification with LTR retrotransposons and DNA transposons as the main sources of the discrepancies (Additional file 1, Fig. S1). DNA transposons and LTRs were predominant in the DeepTE classification with 50% of DNA transposons identified by RepeatModeler2 classified as LTR and vice versa (Additional file 1, Fig. S1A). Furthermore, LINEs and Helitrons appeared underrepresented in DeepTE. RFSB reclassified less than 25% of DNA transposons as LTRs but recognized the majority of RC/Helitrons as LTRs and the rest as SINEs (Additional file 1, Fig. S1B). Inconsistent classification of repetitive elements could likely be due to substantial sequence divergence or nested insertions. In order to compare the classifications on a cleaner data set, we focused on single exon transcripts which completely overlap TEs and are therefore our best candidates for active transposons. The corresponding sequences should be less degenerated and the probability of nested insertions should be minimized. Based on a manual inspection of classifications of 200 randomly chosen sequences, we found that in 73.3% of cases, all three methods agreed on the TE class (DNA transposon/retrotransposon). In addition, RepeatModeler2 showed the lowest error rate when compared to the other two classification methods (Additional file 1, Fig. S2). Therefore, we decided to use the RepeatModeler2 classifications for further analysis.

### The distribution of TE across chromosomes depends on the class

**Fig. 2:**
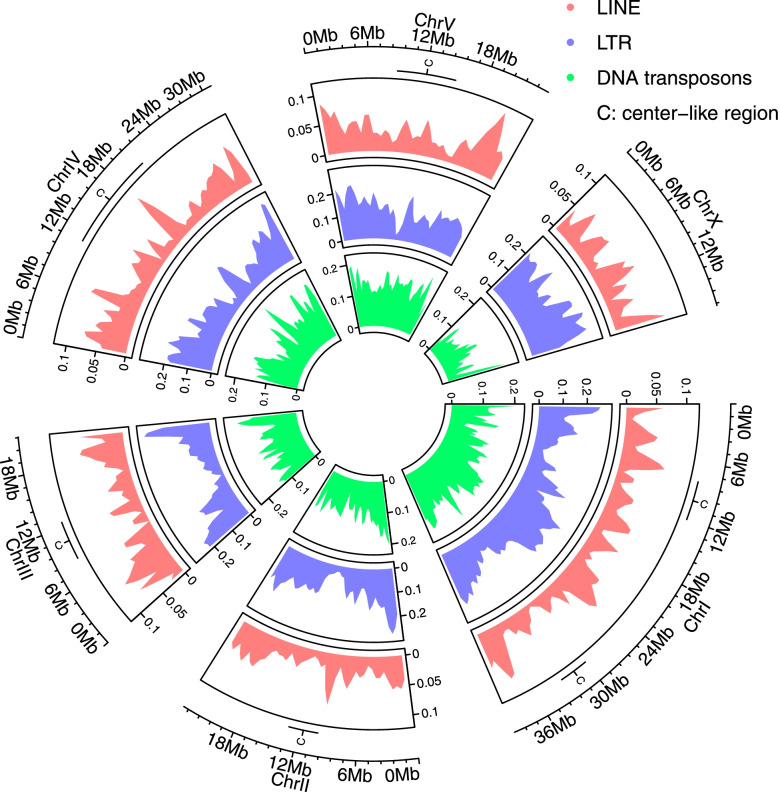
Distribution of DNA transposons, LINEs and LTRs as identified by RepeatModeler2 across the chromosomes of *P. pacificus* revealed TE enrichment in the arms of the autosomes and a depletion in the chromosome centers

The general distribution of repeats across chromosomes has been analyzed previously showing lower repeat density at chromosome centers [14]. Note that that *P. pacificus* chromosome I has two center-like regions that are also defined by high gene density and low sequence diversity. To investigate the chromosomal distribution of the different TE subclasses, we calculated the fraction of coverage by DNA transposons, LINEs and LTRs per 5-kb window. In chromosome I DNA transposons were roughly evenly distributed in contrast to LINEs and LTRs which showed higher TE densities at the arms and the middle of the chromosome I (Fig. 2). LTRs and LINEs followed a similar distribution pattern in chromosomes II, III and V, with a noticeable decline in center-like regions and enrichment at the chromosome arms (Fig. 2). Enrichment towards the chromosomal arms was also observed in chromosome IV for all three types of elements. The distribution of DNA transposons, LTRs and LINEs was more even on chromosome X. Furthermore, we examined the distribution of the repeat datasets produced by RepeatModeler2, RED and Tantan. The selected datasets represent the three methods that masked the highest percentage of the genome. RepeatModeler2 and RED exhibited almost identical distribution across all chromosomes (Additional file 1, Fig. S3). In summary, the lower repeat density at the chromosome center holds true for LINEs and LTRs while DNA transposons exhibit mostly a more even distribution.

### DNA transposons and LINEs show evidence of expression as single-exon transcripts

**Fig. 3:**
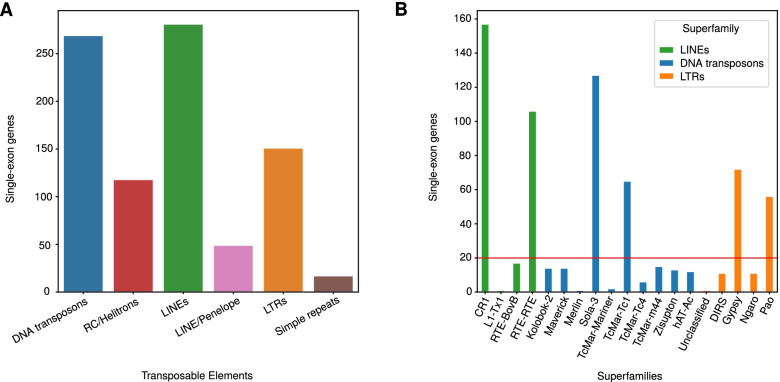
Expression of TEs and simple repeats. **A** Investigation of single-exonic transcripts fully overlapping repeat sequences revealed the best candidates for active transposons. **B** Further analysis identified the CR1, Sola-3, RTE, TcMar-Tc1, Gypsy and Pao superfamilies as the most overrepresented superfamilies among DNA transposons, LINEs and LTRs with 100% exon coverage in single-exon transcripts. The threshold for determining the most abundant superfamilies was set at a coverage of at least 20 single-exon transcripts

To examine the expression of TEs and simple repeats, we identified single-exon (SE) genes from an existing transcriptome assembly [35]. We initially investigated the repeats fully overlapping SE genes to gather evidence for active transcription of TEs. In total, 14% of single exon genes have their exons fully covered by TEs. Out of a total of 897 TE fully covering SE genes, 281 were LINEs, 269 DNA transposons, 151 LTRs, 118 RC/Helitrons, 49 Penelope elements and only 29 Simple repeats (Fig. 3A). To determine the best candidates for active TEs, we set a cutoff of at least 20 single-exon genes covered by a single superfamily. Among the LINEs with evidence of expression, the CR1 and RTE superfamilies were the most abundant while the most overrepresented DNA transposons superfamilies were Sola-3 and TcMar-Tc1 (Fig. 3B). The Gypsy and Pao superfamilies were the two most abundant contributors regarding LTRs with evidence of transcription (Fig. 3B). Thus CR1, Sola-3, RTE, TcMar-Tc1, Gypsy and Pao are the best candidates for active transposons in *P. pacificus*.

### Protein-coding genes of all age classes exhibit contributions from TEs

**Fig. 4:**
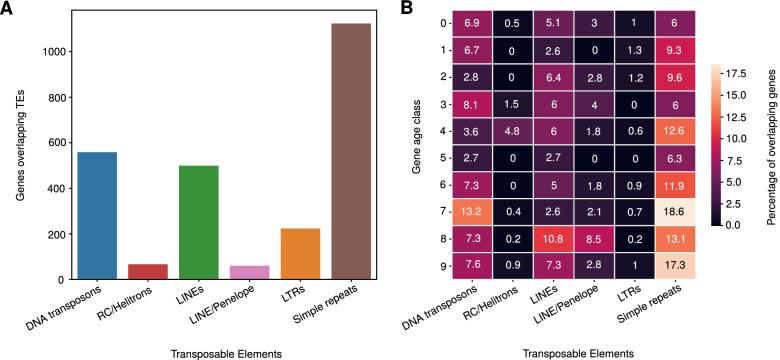
**A** Coverage analysis of repeat sequences with the current gene annotations showed that protein-coding genes are enriched with simple repeats contrary to single-exon genes. **B** Tandem repeats remained the most overrepresented repeat sequences after inspection of protein-coding genes spanning the nine gene age classes (see *Methods*). The fraction of genes overlapping simple repeats gradually increased from 6% in *P. pacificus* exclusive genes to 17.3 and 18.6% of genes conserved with *M. japonica* and *P. fissidentatus*, respectively with a noticeable depletion of all repeat sequences in age class five

Previous analysis in *P. pacificus* has shown that repeats can lead to homology detection failures, thereby contributing to the classification of coding sequences as orphan genes [19]. Furthermore, around half of primate-specific orphan genes show traces of TEs [18]. To assess the contribution of TEs and repeats in protein-coding genes, we screened for overlaps between TEs/repeats and the complete gene annotation for *P. pacificus* [16]. For this purpose, we changed the overlap threshold to a minimum of 50% exon coverage by repeats. Simple repeats accounted for 1126 out of 2569 genes overlapping repeats (Fig. 4A). On the contrary, TEs did not exhibit a high number of overlaps as was the case with SE genes (Fig. 4A). The trend for the overrepresented DNA transposon superfamilies was similar to SE genes with Sola-3 and TcMar-m44 being heavily overrepresented (Additional file 1, Fig. S4). Compared to LINE-associated exons in SE genes, the CR1 superfamily remains the predominant one.

To test whether transposons disproportionately contribute towards new gene formation, we quantified the overlap between repeat elements and protein-coding genes across different age classes (minimum 50% of an exon). These age classes were defined based on phylostratigraphic analysis of ten diplogastrid genomes that form a ladder-like phylogeny [36]. Genes were assigned to age classes based on the presence of BLASTP hits (e-value< 0.00001) in the most distantly related genome, with *P. pacificus*-specific genes being assigned to age class 0 and genes with homologs in the genome of *M. japonica* being assigned to age class 9. The majority of genes in all age classes did not show an overlap with TEs or simple repeats (Fig. 4B). We found that simple repeats were overrepresented across all age classes with the oldest age classes exhibiting the highest percentage.

We performed a Gene ontology (GO) term overrepresentation analysis for the oldest genes with repetitive sequences. Specifically, we employed the David webtool (https://david.ncifcrf.gov/summary.jsp) to test for enriched GO terms in *C. elegans* orthologs of genes with simple repeats or low complexity regions against a background set of all *C. elegans* orthologs. This identified ‘nucleus’ (GO:0005634, corrected *P* = 1.3 × 10^− 13^), ‘nucleic acid binding’ (GO:0003676, corrected *P* = 3.2 × 10^− 8^) and ‘DNA binding’ (GO:0003677, corrected P = 1.3 × 10^− 8^) as the most significantly enriched terms, followed by terms like ‘locomotion’ (GO:0040011, corrected *P* = 1.5 × 10^− 5^) and ‘hermaphrodite genitalia development’^1^ (GO:0040035, corrected *P* = 7.7 × 10^− 5^). One example of these genes is the ortholog of *C. elegans dpy-22*, which functions as a transcriptional coactivator [37]. Multiple simple repeats span protein-coding exons of the *P. pacificus* ortholog. Protein translations of these repetitive sequences result in a glutamine-rich C-terminal region that is also found in *C. elegans dpy-22* (Additional file 1, Fig. S5).

### Horizontal gene transfer has led to an ancient invasion by DNA transposons into diplogastrid genomes

**Fig. 5:**
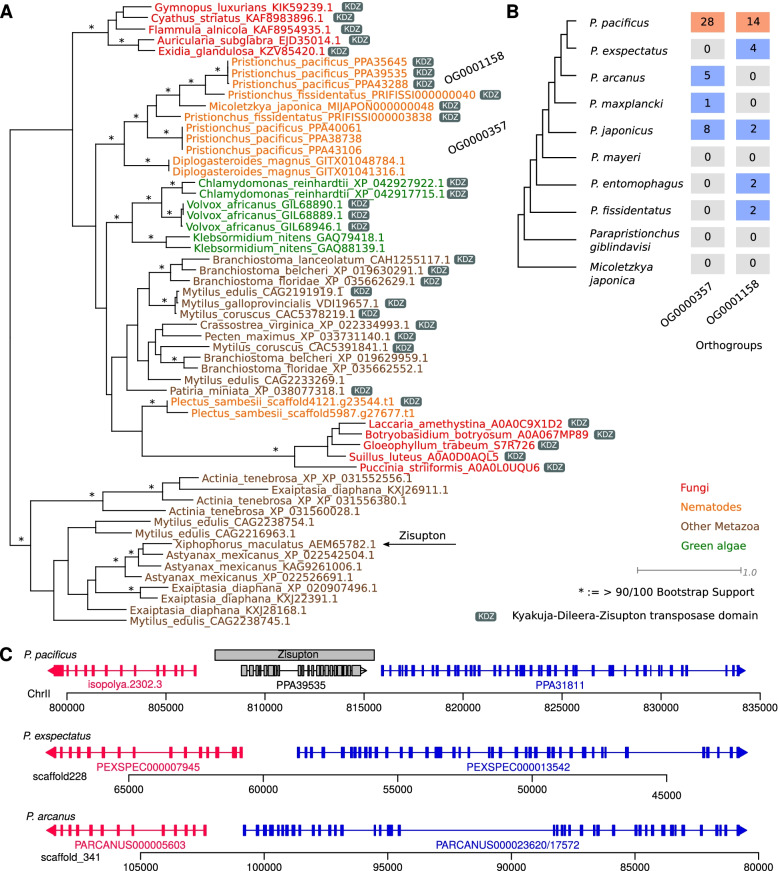
**A** Homology searches of putative Zisupton transposons in *P. pacificus* identified most closely related sequences in other metazoan phyla as well as green algae and fungi. The phylogenetic tree was reconstructed from a protein alignment of the Kyakuja-Dileera-Zisupton transposase domain (PF18758). The distinct grouping of the two orthologous families in *P. pacificus* from a homolog in the nematode *Plectus sambesii* suggests independent horizontal gene transfers. **B** Orthology analysis across ten diplogastrid genomes elucidates the recent evolutionary history of Zisupton activity. The plot shows the numbers of orthologs for both families across the schematic phylogeny. While the orthologous family OG0000357 seems to have arisen only recently, both families showed rapid expansions in the *P. pacificus* lineage. **C** Syntenic analysis identified evidence of recent transposon activity in *P. pacificus*. The plot shows gene structures of syntenic regions in *P. pacificus*, *P. exspectatus*, and *P. arcanus*. Orthologous genes are indicated by identical colors. While synteny is conserved between the two outgroup species, the *P. pacificus* genome shows a specific insertion of a putative Zisupton transposon

The comparison of transposon annotations with current gene models (Fig. 4A) revealed a large number of DNA transposons (Fig. 3). Fifty-eight of these sequences correspond to regions that were classified as Zisupton transposons by RepeatModeler2. Zisupton denotes a class of multi-exonic DNA transposons that were initially characterized in fishes, but are also present in fungi and algae, which suggested horizontal gene transfers [38]. In *P. pacificus*, we identified two orthologous gene families comprising more than 40 genes that overlap annotated Zisupton regions. Similar to their homologs in fishes, the corresponding proteins are up to 1400 amino acids in length. BLASTP searches against the NCBI nr database identified the best hits in green algae, fungi and other metazoans such as lancelets (*Branchiostoma floridae*), sea stars (*Patiria miniata*) and mussels (Fig. 5A). Complementary BLASTP searches against 147 nematodes (excluding diplogastrids) on WormBase ParaSite (version WBPS16) [39] identified only hits (e-value < 0.001) in the nematode *Plectus sambesii* [40]. However, phylogenetic analysis indicated that Zisupton sequences from *Pristionchus* and *Plectus sambesii* do not form a monophyletic clade. This suggests that they derived from independent horizontal gene transfers. More detailed analysis of the two orthologous families shows that one family (OG000357) has arisen only recently in the *Pristionchus* genus whereas the second family (OG00158) seems to be much older as orthologs exist in almost all *Pristionchus* species. Additional BLASTP searches could identify a homologous sequence in *Micoletzkya japonica* and *Diplogasteroides magnus* (Fig. 5A), which indicates that the initial invasion presumably occurred in the diplogastrid family. Moreover, both orthologous gene families have undergone recent expansions in the *P. pacificus* lineage (Fig. 5B). For both recently expanded orthogroups, we identified a core region of nearly perfect sequence identity. This core region spanned 8099 and 8870 nucleotides in the orthogroups OG00158 and OG000357, respectively. The corresponding protein products differed substantially in their protein length and gene structure, ranging from 1166 amino acids and 18 exons for OG00158 to 1367 amino acids and 31 exons for OG000357 (Additional file 1, Fig. S6A). In addition, alignment of the 5′ and 3′ non-coding regions showed evidence of terminal inverted repeats (Additional file 1, Fig. S6B) but we could not detect any target site duplications.

To identify more direct evidence of recent transposon activity, we screened conserved syntenic regions between the three most closely related *Pristionchus* species for a *P. pacificus*-specific insertion of a Zisupton sequence. Figure 5C shows an example of a *P. pacificus*-specific Zisupton insertion in a conserved syntenic region between *P. exspectatus* and *P. arcanus*. Thus, our analysis suggests that horizontal gene transfer has led to an ancient invasion of DNA transposon into the diplogastrid family and these transposons have undergone a recent wave of increased activity along the *P. pacificus* lineage.

### There is no general trend of higher repeat content in *P. pacificus*

**Fig. 6:**
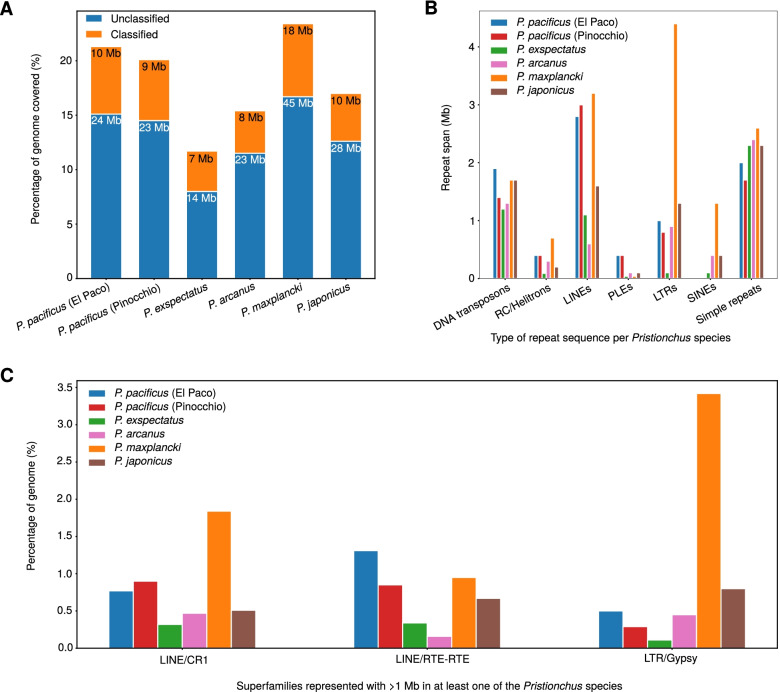
**A** The bars show the percentage of the genome assemblies that could be annotated as repetitive regions by Repeatmodeler and Repeatmasker. **B** The plots show the distribution of classified repetitive elements across the *Pristionchus* genomes. **C** Superfamilies spanning at least 1 Mb in any *Pristionchus* genome are shown for different species

The previous analysis showed an increased activity of putative DNA transposons in the *P. pacificus* lineage. This lineage also represents a transition of the reproductive mode from a gonochoristic ancestor (females, males) to androdioecious species (hermaphrodites, males). One consequence of the evolution of hermaphroditism in nematodes is the ability to reproduce by selfing. Previous studies demonstrated that the degree of selfing can impact the activity of TEs [41, 42]. To test whether *P. pacificus* shows evidence for a generally increased transposon activity, we compared the repeat content in *P. pacificus* with four close relatives, *P. exspectatus*, *P. arcanus*, *P. maxplancki*, and *P. japonicus* [36]. For better comparability with the short-read assemblies of these gonochoristic species, we have included an alternative short read assembly of *P. pacificus* (version Pinocchio) in this comparison (Fig. 6). This analysis shows that *P. pacificus* has a higher repeat content than its closest relatives *P. exspectatus* and *P. arcanus* (Fig. 6A). However, the genome of *P. maxplancki* has the overall highest repeat content (Fig. 6A). Further inspection of transposon classes shows that only LINE elements (CR1 and RTE-RTE) are much more abundant in *P. pacificus* when compared to *P. exspectatus* and *P. arcanus* (Fig. 6B, C). Moreover, SINE elements which appear to be missing in *P. pacificus* are present in the other genomes (Fig. 6B). The more distantly related genome of *P. maxplancki* has much higher levels of LTRs and SINEs, when compared to *P. pacificus*. Thus, the androdioecious genome of *P. pacificus* does not generally have the highest content of repeats and TEs.

## Discussion

How well do we know our genomes? Certainly, we have gained tremendous knowledge over the last twenty years after the sequencing of the first metazoan genomes. With constantly developing technologies, genome sequencing and functional genomic studies allowed us to identify disease associations, to gain evolutionary insights, and to characterize various mechanisms of gene regulation. However, even for an extensively studied genome such as *C. elegans*, more than 40% of genes lack functional annotation [43]. For a more exotic model organism such as *P. pacificus*, only dozens of genes have been experimentally characterized [44–46] and the inference of functional annotations based on homology is hampered by the fact that around one third of genes are classified as orphan genes without detectable homologs outside the diplogastrid family [19]. Hereby, we are ignoring the fact that largest parts of the genomes are not protein-coding. Thus, there still seems to be a long way to go before we understand how gene expression levels are regulated and which parts of the genomes are functional and which not. Ironically, with more and more sequencing data, it seems to become less clear how we define genes in the first place and what is biological function [47–49].

The primary objective of the current study was to extend our knowledge of the *P. pacificus* genome by characterizing its repetitive regions. In order to capture the full diversity of repetitive sequences ranging from low complexity regions to DNA transposons and retrotransposons, we applied multiple different computational approaches. We would argue that the large-scale differences in their predictions are mostly due to their specific objectives for identifying different classes of repetitive elements. However, these differences together with problems in classification also suggest that comprehensive annotation of repetitive elements in divergent genomes is not straightforward. In the end, we focused on the predictions by RepeatModeler2, because it is a unified approach to identify all types of repetitive elements, it annotated a similar fraction of the *P. pacificus* genome in comparison with *C. elegans*, it is able to classify TEs, and it also showed fewest classification errors in our evaluation. We then used these annotations to screen for evidence of active transposons in available transcriptome data. Future analysis of divergent *P. pacificus* strains could be used to support that the transposon activity is not only limited to the transcriptional level but actually results in transposition events.

A second major objective of our study was to investigate the impact of repetitive sequences and TEs in the formation of novel genes. Numerous studies have shown that transposons show substantial contributions to novel genes and that individual domains can be coopted to serve new functions in the host organism [50]. While we do not see an obvious signal for an enrichment of TE derived sequences in very young genes, there is a consistent fraction of roughly 10–20% with contributions of transposon-derived sequences across all age classes. The strongest signal seems to be the large fraction of simple repeats in old gene classes. We would speculate that these low complexity regions form structural motifs such as coiled coils where the whole region is constrained to exhibit specific structural properties but little selection acts on individual amino acids [51]. Finally, we identified homologs of a class of DNA transposons that is absent in *C. elegans* and most other nematodes. These sequences likely invaded the ancestor of diplogastrid nematodes by horizontal gene transfer from another eukaryote. Horizontal gene transfer of transposons seems to be frequent and has been described in another type of transposons even in *P. pacificus* [52, 53]. The Zisupton elements are unusual in a way that they are multi-exonic which makes them superficially look like typical protein-coding genes. In *P. pacificus*, we found dozens of instances of Zisupton homologs, which seems to be a result of a recent burst of transposon activity after the switch to hermaphroditism. As these sequences are technically taxon-restricted orphan genes, they constitute another example that in addition to sequence divergence, and de novo formation, also horizontal gene transfer contributes to the emergence of novel genes [54].

## Materials and methods

### De novo repeat detection

We used eleven tools for de novo repeat identification in *Pristionchus pacificus* in order to represent the diversity of approaches with regard to the type of repeat they detect. The methods used for locating TE can be classified in library-based, signature-based, learning-based, homology-based, de novo and consensus while the detection of tandem repeats includes library-based, learning-based and de novo methods [20]. We utilized RepeatMasker (A.F.A. Smit, R. Hubley & P. Green RepeatMasker at http://repeatmasker.org) with *C. elegans* as a reference from the library-based methods as well as the standalone version of LTRharvest [21] with the index produced by Tallymer [22] and MiteFinderII [23] (version 1.0.006, parameters: -threshold 0.6) as signature-based programs. From the de novo detection methods available, we applied Tallymer [22] (genometools suite version 1.6.1, Suffixator: -dna -pl -tis -suf -lcp -v -parts 4, Tallymer occratio: -scan -output unique relative -minmersize 8 -maxmersize 20, parameters for -scan -mersize 19 -minocc 40 -counts -pl), RED [20] (version 2.0, parameters: -frm 2), mReps (version 2.6, parameters: -res 5 -exp 3.0), Tandem Repeat Finder [24] (version 4.09, parameters optimized for *C. elegans*: 2 5 5 80 10 402,000 -f -d -m) and TANTAN [25] (version 23, parameters: -r 0.02). We also selected MsDetector [26] (MsDetectorOptimized64, version 1.2) for locating tandem repeats and RepeatModeler2 [27] as the consensus method for both TE and TR detection (version 2.0.1, parameters: -LTRstruct). Low-complexity regions were identified using Dustmasker and sDust [28] with default parameters. We fragmented the nematode genome in consecutive 1-kb windows with the BEDTools suite [55] (option: make windows) in order to count the number of TEs and tandem repeats overlaps per 1 kb for each class. We created a binary vector based on the coverage of the 1-kb windows by each repeat finder and performed hierarchical clustering (Euclidean distance, complete-linkage). For the rest of the analysis, SINEs were excluded as RepeatModeler2 did not assign any of the identified TE to this order.

### Evaluation of classification algorithms

To test whether TEs labelled as “Unknown” by RepeatModeler2 could be classified with DeepTE [33] and the RFSB classifier from transposonUltimate [34], we tested both classifiers with the labelled TE from RepeatModeler2. DeepTE was used with default parameters for metazoans (−sp M) and RFSB was run on -mode classify. Both methods were compared to RepeatModeler2. In addition, single exon transcripts from the transcriptome assembly were overlapped with annotated repeats from RepeatModeler2. Under the assumption that such transcripts represent active transposons, classification should be easier as these sequences should be less degenerated and nested insertions should not occur. We therefore chose a small subset of 200 single exon transcripts for manual comparison of classification accuracy between RepeatModeler2, RFSB, and DeepTE. The data set comprised 45 transcripts, where we could annotate protein domains in complete or partial ORFs using the hmmsearch program HMMER package (version 3.3, e-value< 0.001) with the Pfam database (version 3.1b2) [56, 57].﻿ The remaining transcripts were randomly chosen. If available, we used protein domain information as an additional source to classify a putative TE. This was done according to the classification scheme proposed by Wicker et al. (2007) [5]. If no protein domain information was available, classification was done based on the majority vote between all three methods.

### Distribution of TEs across the *P. pacificus* genome

To investigate the distribution of the three most abundant TEs (LINEs, DNA transposons and LTRs) in *P. pacificus* we divided the genome in 5-kb continuous windows. Subsequently we calculated the coverage by each type of TE as the fraction of window length using the BEDTools coverage option. The distribution of TEs across the genome was determined with the circlize package from R (function: circus.genomicDensity()).

### Phylostratigraphic analysis

Protein coding genes from *P. pacificus* (version El Paco 3) were classified into age classes (phylostrata) based on the presence of most distant homologs in the phylogenomic data set of nine other diplogastrid nematodes. Age classes 0 defined *P. pacificus* specific genes and older age classes were defined based on the presence of homologs in *P. expectatus* (Age class 1), *P. arcanus* (2), *P. maxplancki* (3), *P. japonicus* (4), *P. mayeri* (5), *P. entomophagus* (6), *P. fissidentatus* (7), *Parapristionchus giblindavisi* (8), and *Micoletzkya japonica* (9). Homologs were identified based on one-directional BLASTP searches using the *P. pacificus* proteins as queries (version 2.10.1, e-value< 0.00001).

### Expression analysis

The strand-specific transcriptome assembly of the *P. pacificus* reference strain PS312 (European Nucleotide Archive: HAKN01000000, [35]) was aligned to the *P. pacificus* genome assembly (version: El Paco [14]) with the help of the exonerate est2genome program (version 2.2.0, [58]). Subsequently, the PPCAC pipeline (version: 1.0) was adjusted to select one representative transcript per 100-bp window without any restriction on exon number or protein sequence length [59]. This resulted in a set of 48,605 non-redundant sequences with evidence of active transcription. In addition, we obtained the current set of gene annotations for *P. pacificus* (version: El Paco gene annotations 3, [16]). For the expression analysis we only selected the TEs classified by RepeatModeler2. We filtered the transcriptome assembly [35] to select only genes with a single exon and used the BEDTools intersect option (parameters: -f 1, −wb) to identify the TEs fully overlapping exons. We created a non-redundant transcriptomic dataset by merging isoforms of the same gene, excluded single-exon genes and searched for TEs covering at least 50% of the exons. Furthermore, we checked the exon overlap distribution for each TE class with the latest *P. pacificus* annotation [16] (minimum 50% coverage of the exon by a TE) and the phylostratigraphic data of *P. pacificus* and nine closely related diplogastrids [36].

### Comparative genomic analysis of putative Zisupton sequences

Putative Zisupton transposons in *P. pacificus* were extracted as gene models [16] that are located in regions that were annotated as Zisupton DNA transposons by RepeatModeler2. The corresponding protein sequences were searched by BLASTP (e-value < 0.001) against the NCBI nr database, against four diplogastrid genomes from Casasa et al. [60], against nine other diplogastrid genomes on http://www.pristionchus.org, and against 147 nematode genomes (excluding diplogastrids) from WormBase ParaSite (version: WBPS16, [39]). The highest sequence similarity was found in regions that corresponded to the Kyakuja-Dileera-Zisupton transposase domain (PF18758). Representative sequences from major taxonomic groups were compiled into a fasta file. This set of sequences was complemented by the original Zisupton sequence [38] and the most closely related sequences from the PF18758 in the Pfam database. A multiple sequence alignment was generated by the MUSCLE aligner (version 3.8.31, [61]). A Maximum-likelihood tree was computed using the pml, optim.pml and bootstrap.pml functions of the phangorn package in R (version 3.4.4, model=”LG”, optNNi = TRUE, optBf = TRUE, optInv = TRUE [62]). Orthologous clustering of ten diplogastrid genomes ([16, 36]) was done using OrthoFinder (version: 2.5.2 [63]) and conserved syntenic blocks were identified by pairwise gene order alignments between *P. pacificus* and either *P. exspectatus* or *P. arcanus* using the Cyntenator software [64].

## Supplementary Information

**Additional file 1: Fig. S1.** TE classification comparison of DeepTE and RFSB to RepeatModeler2. **Fig. S2.** Manual inspection and comparison of classification between RepeatModeler2, DeepTE and RFSB. **Fig. S3.** Circos plots of TEs identified by RepeatModeler2, RED and Tantan by order. **Fig. S4.** Superfamilies present in the most abundant expressed TEs. **Fig. S5.** Example of simple repeats overlapping a gene conserved between *P. pacificus* and *C. elegans.* **Fig. S6.** Comparison of gene structures for representative genes of both Zisupton-related orthogroups.

## Data Availability

The *P. pacificus* genome is available at NCBI Genbank under the accession number ABKE04000000. Repeat annotations are available at http://www.pristionchus.org/download.

## Abbreviations

CR1: Chicken repeat 1
LINE: Long interspersed nuclear element
LTR: Long terminal repeat
RC: Rolling circle
RTE: Retrotransposable element
SE: Single exon
TE: Transposable element

## Footnotes

1 As was pointed out by an anonymous reviewer and was also confirmed by other colleagues, hermaphroditic nematodes have female genitalia.
