# Supplementary material for: Analysis of repeat elements in the Pristionchus pacificus genome reveals an ancient invasion by horizontally transferred transposons
Source: BMC Genomics. 2022 Jul 19;23:523. doi: 10.1186/s12864-022-08731-1 (PMC9297572; doi:10.1186/s12864-022-08731-1)
Supplement: Supplementary file 1 — Additional file 1: Fig. S1. TE classification comparison of DeepTE and RFSB to RepeatModeler2. Fig. S2. Manual inspection and comparison of classification between RepeatModeler2, DeepTE and RFSB. Fig. S3. Circos plots of TEs identified by RepeatModeler2, RED and Tantan by order. Fig. S4. Superfamilies present in the most abundant expressed TEs. Fig. S5. Example of simple repeats overlapping a gene conserved between P. pacificus and C. elegans. Fig. S6. Comparison of gene structures for representative genes of both Zisupton-related orthogroups. [file 12864_2022_8731_MOESM1_ESM.pdf]

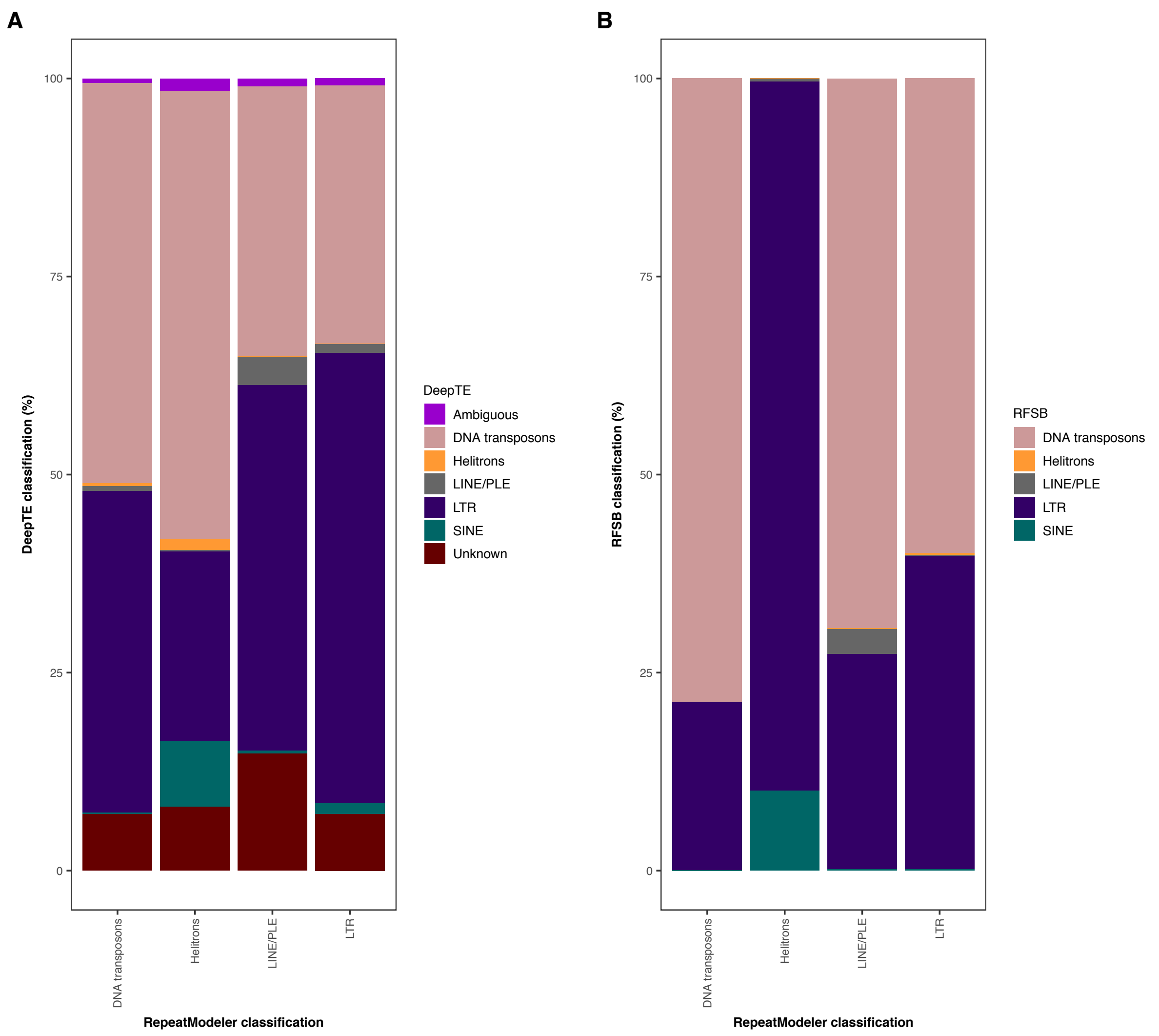

Fig. S1 (A) Reclassification of the RepeatModeler2-annotated TEs using DeepTE resulted in inconsistencies, particularly when distinguishing LTRs from DNA transposons. The same was observed with LINEs which are the most abundant TE order in the RepeatModeler2 dataset. **(B)** RFSB was more accurate when classifying DNA transposons but showed disagreement in more than 50% of LTRs. LINEs were also underrepresented in the RFSB dataset.

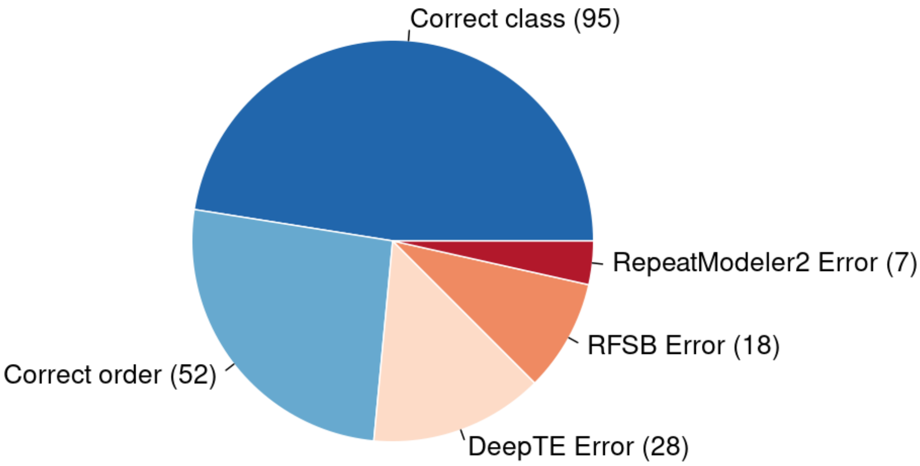

Fig. S2. The manual inspection of 200 transcribed single exon genes that have been classified as transposons shows that all three classification methods agree in 73.5% on the transposon class (DNA transposon or retrotransposon). Correct order indicates correct assignment of order, as defined by Wicker et al. 2007, e.g. LTR, or LINE. RepeatModeler2 appears to have the lowest error rate (3.5%), followed by RFSB (9.0%), and DeepTE (14.0%).

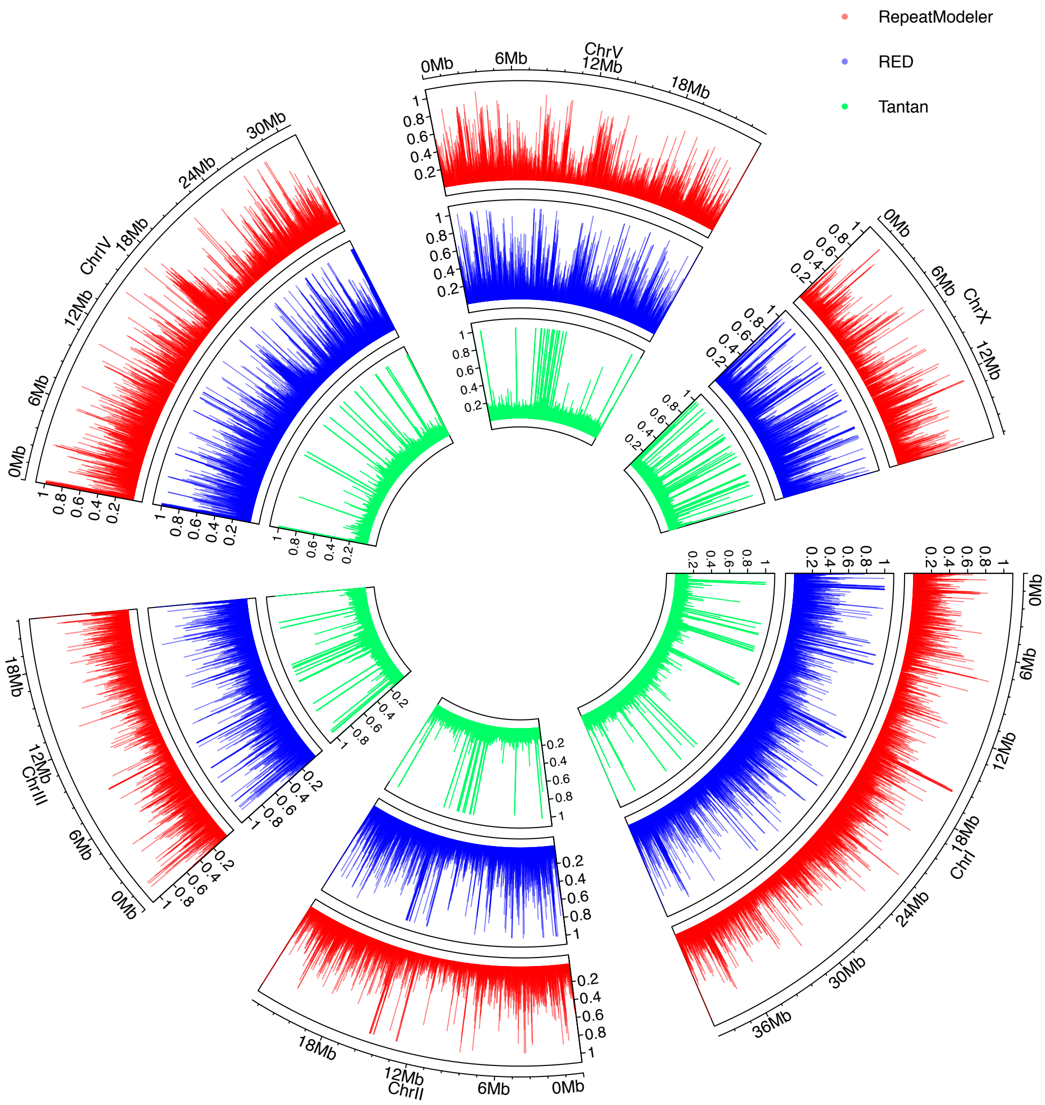

Fig. S3. The distribution of repeat sequences identified by RepeatModeler2 and RED show similar profiles across the *P. pacificus* chromosomes while TANTAN exhibited certain more TE-dense areas in the autosomes.

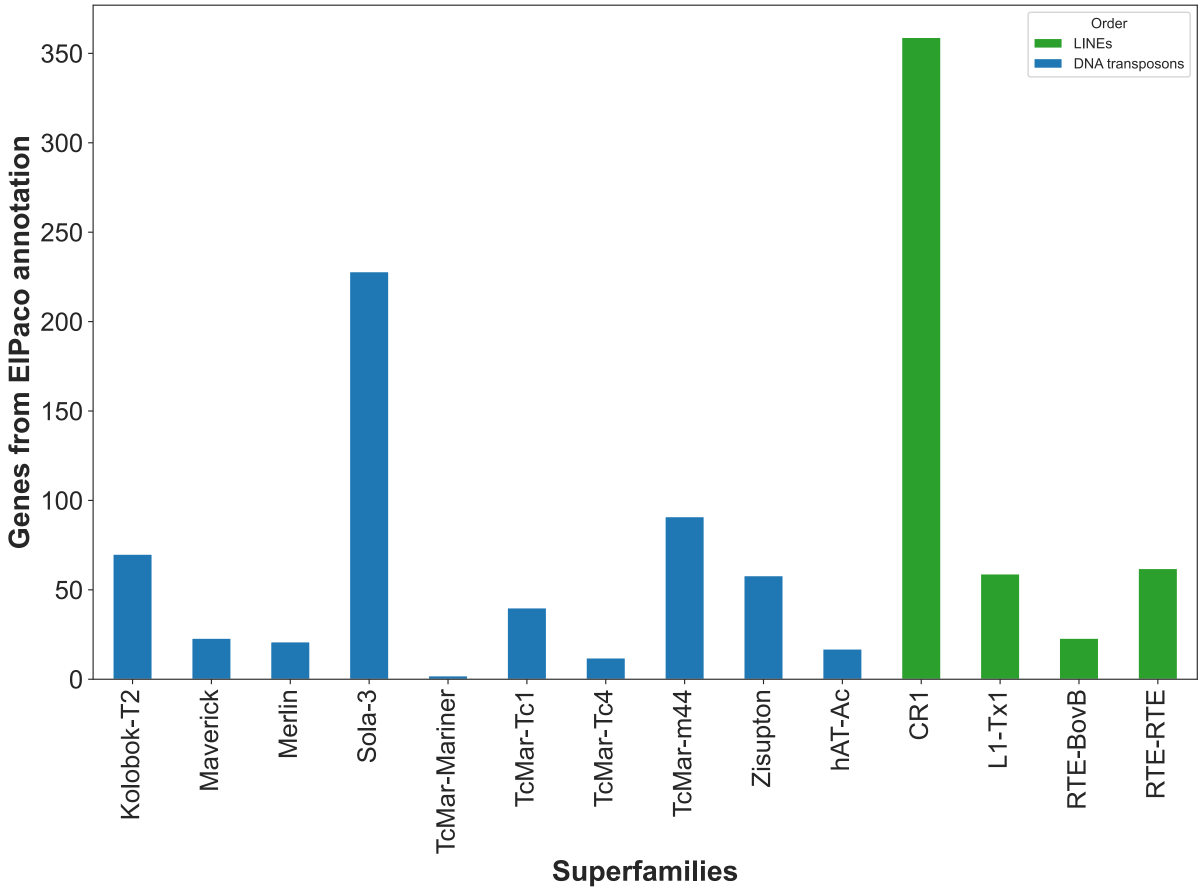

Fig. S4. CR1, Sola-3 and TcMar-m44 were the most overrepresented superfamilies among TEs spanning the exons of protein-coding genes.

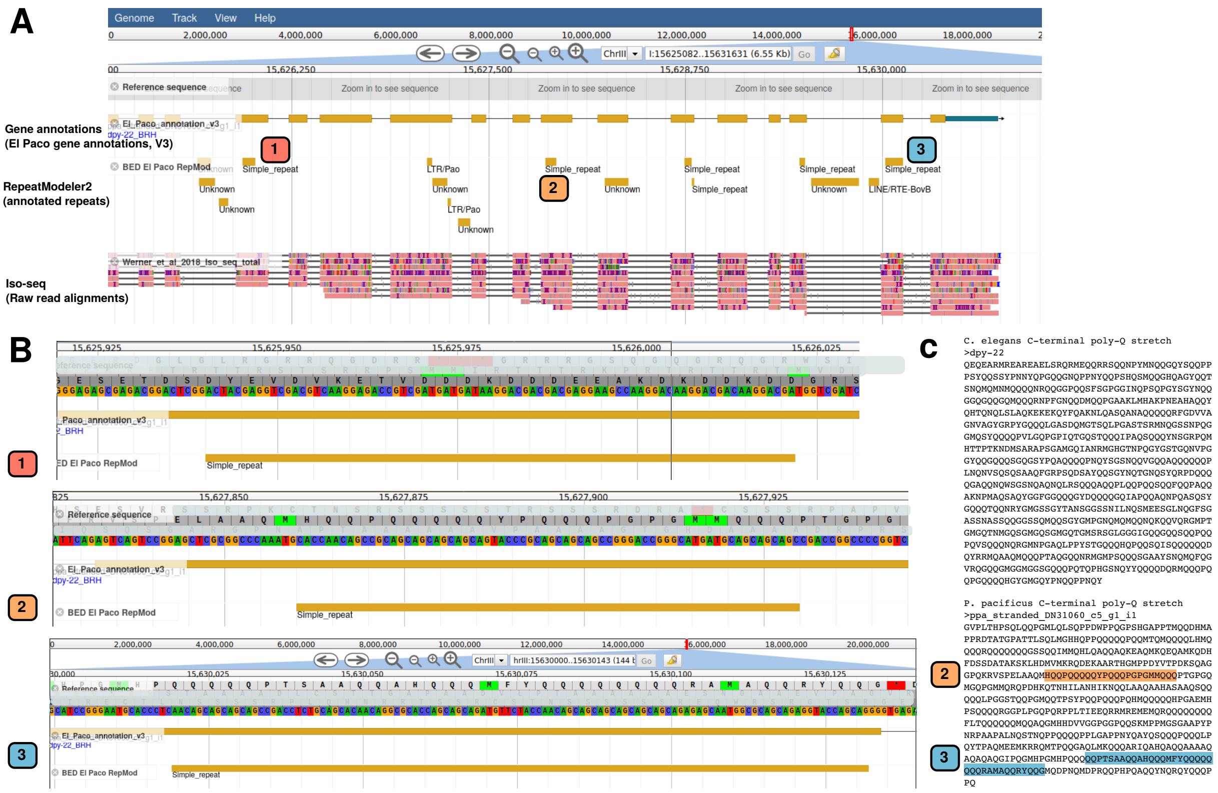

Fig. S5. **(A)** The *P. pacificus* ortholog (ppa_stranded_DN31060_c5_g1_i1) of *C. elegans dpy-22* has multiple simple repeats in protein-coding exons. The screenshots show the genomic locus of the C-terminal region of *P. pacificus dpy-22*. The gene structure is well supported by transcriptomic data (Iso-seq track). **(B)** Selected repeats in the C-terminal region are shown with the repeat sequences and their protein translations. **(C)** The poly-glutamine stretch in the C terminal region is found in *C. elegans* and *P. pacificus*.

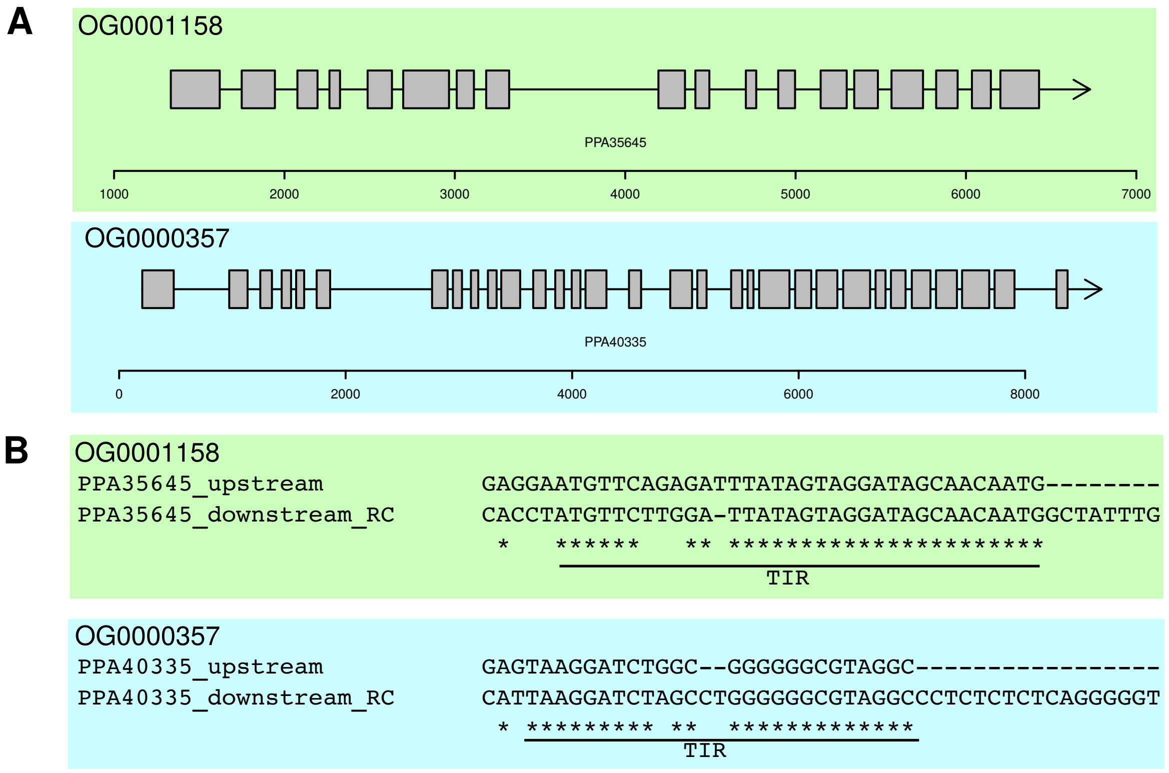

Fig. S6. **(A)** The comparison of gene structures for representative genes of both Zisupton-related orthogroups showed pronounced differences. **(B)** Alignment 5’ and reverse complemented 3’ non-coding regions showed evidence of terminal inverted repeats (TIRs).
